# Appraising the potential of Zr-based biomedical alloys to reduce magnetic resonance imaging artifacts

**DOI:** 10.1038/s41598-020-59247-1

**Published:** 2020-02-14

**Authors:** Anderson Kiyoshi Suzuki, Kaio Niitsu Campo, Eduardo Bertoni Fonseca, Luana Caldeira Araújo, Flávio César Guimarães Gandra, Éder Sócrates Najar Lopes

**Affiliations:** 1 School of Mechanical Engineering, University of Campinas – UNICAMP, 13083-860 Campinas, SP Brazil; 2 Institute of Physics “Gleb Wataghin”, University of Campinas – UNICAMP, 13083-970 Campinas, SP Brazil

**Keywords:** Biomedical engineering, Biomedical materials

## Abstract

This study compared Zr-Mo alloys with commercial metallic biomaterials. It was observed that the Zr-Mo alloys exhibited favourable mechanical properties, particularly the Zr-10Mo alloy, which showed the highest strength to Young’s modulus ratio among all evaluated metals. These alloys also exhibited the lowest magnetic susceptibilities, which are important for magnetic resonance imaging (MRI). However, both Zr- and Ti-based metals yielded comparable artifacts. It was concluded that the magnetic susceptibility must differ considerably to afford significantly improved MRI quality owing to the increased importance of non-susceptibility-related artifacts when comparing materials with relatively similar magnetic susceptibilities.

## Introduction

Metals are still competitive with other classes of materials in the application and development of medical implants in various fields of medicine, such as orthopaedics, neurology, cardiology, and dentistry. Owing to their combined properties, including strength and toughness, and the possibility of employing cost-effective manufacturing processes, metals are typically the best biomaterial choice when compared to ceramics and polymers, as well as composites. The use of metallic devices in the human body is usually required for the replacement and stabilization of damaged bone tissues in orthopaedic practice. The classical examples of such applications include temporary plates and screws, and permanent total hip replacements. Other applications include usage in vascular stents, aneurysm clips, pacemakers, dental implants, wire sutures, *etc*.^1–3^.

Despite being similar to Ti alloys, particularly in their physical metallurgy and other properties^4^, Zr-based alloys have received comparatively little attention as biomaterials. Zr and its alloys exhibit excellent biocompatibility, good corrosion behaviour, and favourable mechanical properties^5–11^. Specifically, these alloys are suitable for applications in orthopaedic and dentistry owing to their low Young’s modulus. A major issue associated with using metallic materials in long-term implants is the mismatch between the bone and the employed device. Rigid implants can promote the stress shielding effect and cause bone resorption, strongly affecting the medium- to long-term quality of the surgical intervention^12^. Accordingly, alloys with a low Young’s modulus have been targeted for this type of application^13,14^. In this regard, Zr alloys are similar to Ti alloys. For instance, Guo *et al*.^10^ reported a Young’s modulus of only 44 GPa for a β-type Zr-12Nb-4Sn alloy, which is relatively close to that of human cortical bone (around 20 GPa^15^) and is similar to as the best results reported for Ti alloys^16^. More recently, increasing interest in Zr and its alloys has been justified by their reduced magnetic susceptibility, which has been claimed to reduce the magnetic resonance imaging (MRI) artifacts^7,10,11,17,18^.

MRI is a powerful non-invasive tool for medical diagnosis that relies on the nuclear magnetic resonance phenomenon. It presents a high spatial resolution and usually outperforms X-ray computed tomography (CT) in contrasting soft tissues; moreover, MRI does not involve an ionizing radiation. Owing to these advantages, MRI is considered as one of the most important techniques for imaging the human anatomy^19^. Nonetheless, it is well known that metallic materials can affect the quality of MR images by introducing artifacts due to perturbation of the magnetic field uniformity^19–23^. The artifacts are dependent on the size, shape, orientation in relation to the magnetic field, and composition of the metallic device^23^. Considering the importance of MRI for medical diagnosis and procedures, as well as the fact that patients with metallic implants have to eventually submit themselves to MRI, it is very important to select and apply materials with good MRI compatibility, which includes properties such as low magnetic susceptibility values^24^. Upon analysis of a few alloys and pure metals, Imai *et al*.^25^ found good linear relationships between the observed artifact volume and the magnetic susceptibility values. Based on such correlations, the authors concluded that low magnetic susceptibility alloys with enhanced MRI compatibility can be developed.

In the present investigation, we focused on the capability of Zr-based alloys in decreasing the MRI artifacts in comparison to well-known metallic biomaterials. Commercially pure (cp) Zr and two binary alloys, Zr-1Mo and Zr-10Mo, were compared to commercially available biomedical alloys: 18Cr-14Ni-2.5Mo stainless steel (ASTM F138), Co-18Cr-6Mo (ASTM F1537), Ti-6Al-4V (ASTM F136), and cp Ti-Gr2 (ASTM F67) (all compositions are in wt.%). Furthermore, the fundamental microstructural and mechanical properties of the Zr-based materials were also studied.

## Results and Discussion

**Figure 1:**
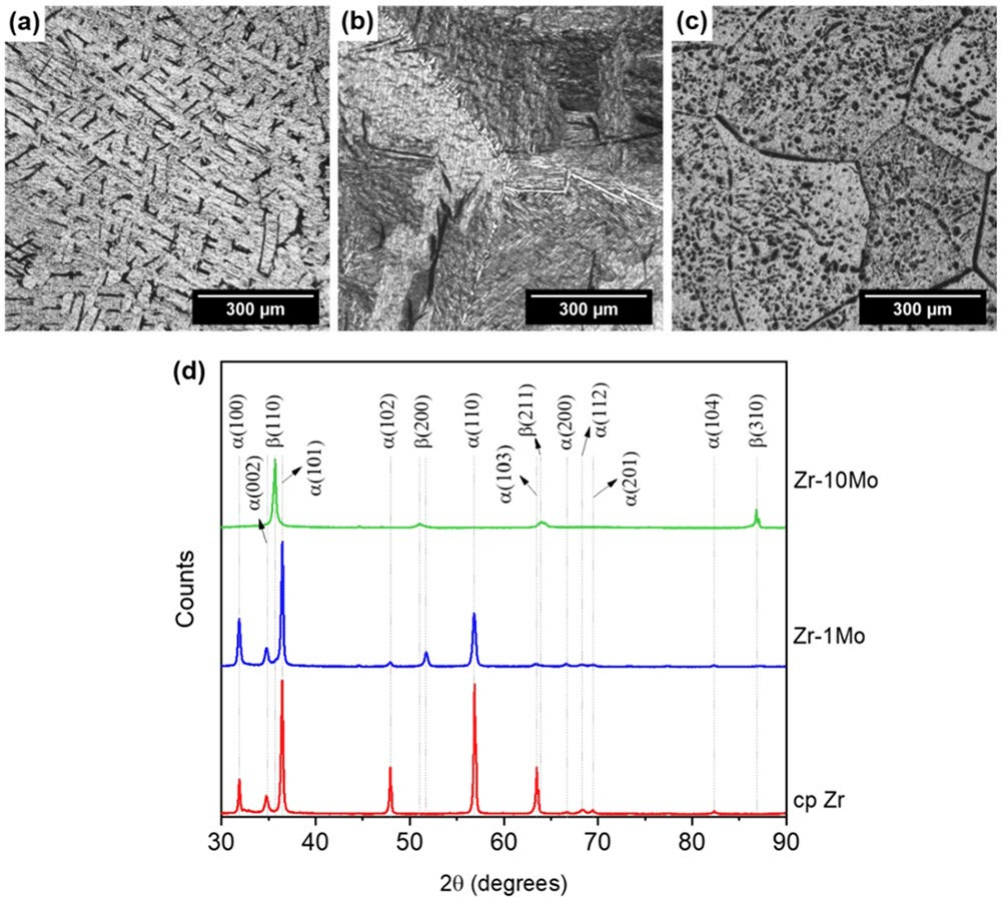
Visible-light microscopy images of the microstructure for the furnace-cooled (**a**) cp Zr, (**b**) Zr-1Mo, and (**c**) Zr-10Mo samples and (**d**) their respective XRD patterns.

Figure 1a–c exhibit the microstructures of the furnace-cooled cp Zr, Zr-1Mo, and Zr-10Mo samples. As the Mo content increased, the volume fraction of the β phase (BCC crystal structure) also increased; this result was expected by the addition of a β-stabilizing element. When 1 wt.% of Mo was added to cp Zr, α phase (HCP crystal structure) colonies became more refined, forming the typical basket-weave structure (Fig. 1b). The presence of a small amount of β phase in this alloy is indicated by the indexed β (200) peak in the XRD pattern of Fig. 1d. Contrarily, the β phase is the major constituent in the Zr-10Mo alloy. As can be seen in Fig. 1c, etched zones at the β-grain boundaries and inside these same grains are mainly related to pits introduced during chemical polishing (conventional and electrochemical polishing were both tried, but neither provided significantly better results), but they can also correspond to small α-phase precipitates. However, even if the α-phase precipitates are present, their volumetric fraction is small, since no peak associated with this specific phase can be observed in the XRD pattern (Fig. 1d).

**Figure 2:**
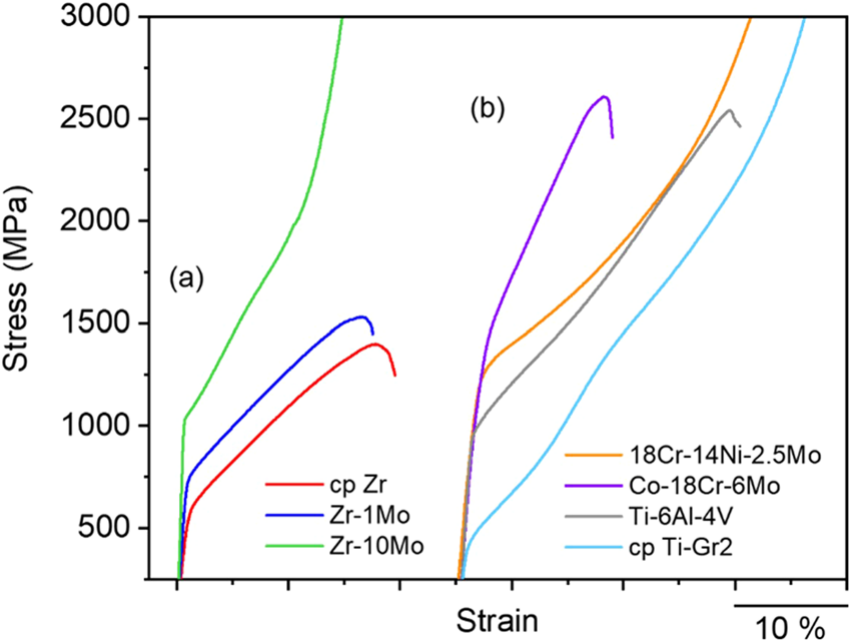
Representative stress-strain curves obtained in the compression tests for the (**a**) furnace-cooled cp Zr, Zr-1Mo, and Zr-10Mo samples, and (**b**) commercial 18Cr-14Ni-2.5Mo stainless steel (ASTM F138), Co-18Cr-6Mo (ASTM F1537), Ti-6Al-4V (ASTM F136), and cp Ti-Gr2 (ASTM F67) samples.

The representative mechanical performance in the compression regime of the Zr-based materials under evaluation can be observed in Fig. 2a. Both strength and ductility increased with Mo content. While fracture occurred approximately at the same strain for Zr and Zr-1Mo, no fracture was observed in the Zr-10Mo alloy; this can be attributed to the high β volumetric fraction in Zr-10Mo, which is typically ductile in the absence of the ω phase. The values obtained for the 0.2% compression yield strength were 566 ± 4, 718 ± 19, and 997 ± 41 MPa for cp Zr, Zr-1Mo, and Zr-10Mo, respectively. The higher strength of Zr-1Mo in comparison to cp Zr is related to both microstructure refinement and solid solution effects, whereas solid solution effects are believed to be the main strengthening mechanism for alloys with increasing Mo content (Zr-1Mo to Zr-10Mo) because the total boundary area decreases when the Mo content is increased. The minor α-phase (if present) precipitation in the Zr-10Mo alloy did not play any significant role in its mechanical behaviour. This alloy exhibited a compression yield strength of 1057 ± 20 MPa and the same ductility as a sample with a full β microstructure, which was obtained after rapid cooling from the β field (water quenching after the solution heat treatment). Interestingly, Zr-10Mo alloy also presented the lowest Young’s modulus (76 ± 1 GPa), while the cp Zr and Zr-1Mo alloys exhibited elastic properties that were significantly higher and similar to each other (the Young’s moduli were 97 ± 1 and 98 ± 1 GPa, respectively). These values are consistent with those reported in the literature^9,26^. Essentially, the α phase presents a modulus of approximately 100 GPa, whereas the values observed for the β phase tend to be lower. For instance, Zhou *et al*.^9^ obtained Young’s modulus values of 99.5, 98.8, and 73.2 GPa for cp Zr, Zr-1Mo, and Zr-10Mo, respectively. Although they were evaluated in the solution-treated condition, analogous phases were found; in this case, the hexagonal martensite α’ was confirmed instead of the original α phase in Zr-1Mo, and a full β microstructure was obtained for Zr-10Mo. In comparison with commercial metals that are typically applied as biomaterials in the human body (Fig. 2b), Zr and its alloys appear to exhibit a good balance between strength and ductility, at least under the conditions examined in this study. Accordingly, from the mechanical properties standpoint, it is reasonable to conclude that Zr-based metals can compete with the current materials. The Zr-10Mo alloy is particularly important owing to its lower Young’s modulus compared to cp Ti-Gr2, which has the lowest value among the commercial metals evaluated (between 103–107 GPa^27^). Furthermore, because of its enhanced mechanical performance, Zr-10Mo also possesses the highest ratio of compression yield strength to Young’s modulus [~13.1 × 10^−3^ against ~8.9 × 10^−3^ for the Ti-6Al-4V alloy (second largest ratio)], which is extremely beneficial for use in high strength medical implants with relatively low levels of rigidity.

**Figure 3:**
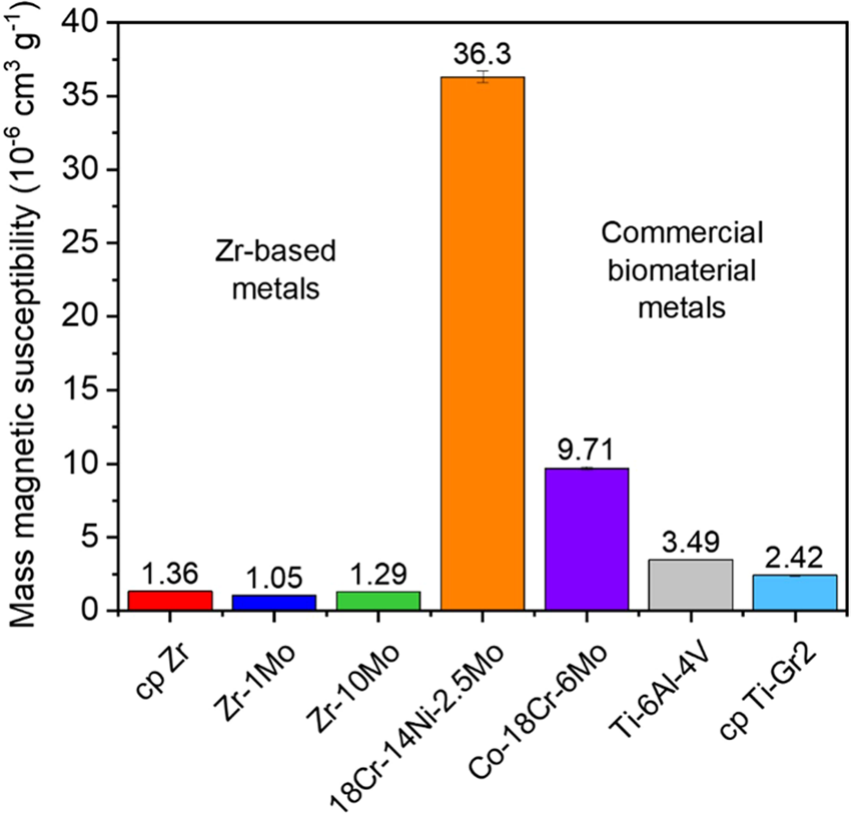
Mass magnetic susceptibility of the furnace-cooled cp Zr, Zr-1Mo, and Zr-10Mo samples in comparison to commercial 18Cr-14Ni-2.5Mo stainless steel (ASTM F138), Co-18Cr-6Mo (ASTM F1537), Ti-6Al-4V (ASTM F136), and cp Ti-Gr2 (ASTM F67) samples.

The magnetic susceptibilities of the furnace-cooled cp Zr, Zr-1Mo, Zr-10Mo together with the other metallic biomaterials are shown in Fig. 3. Notably, the Zr-based materials exhibited the lowest values, varying from 1.36 10^−6^ cm^3^ g^−1^ for cp Zr to 1.05 10^−6^ cm^3^ g^−1^ for Zr-1Mo, and finally to 1.29 10^−6^ cm^3^ g^−1^ for Zr-10Mo. The observed trend is in good agreement with the results published by Suyalatu *et al*.^28^. As the α phase is dominant in cp Zr and Zr-1Mo alloy, their dissimilar susceptibilities can be explained on the basis of their chemical composition, as the magnetic susceptibility of Mo is lower than that of Zr (0.75 and 1.32 10^−6^ cm^3^ g^−1^, respectively^29^). However, a simple and direct relationship based on the Mo content could not be established. Moreover, a further increase in the Mo content did not cause the magnetic susceptibility to diminish, but instead to increase. This can be mainly attributed to the β phase, which has been reported to possess the highest magnetic susceptibility among those typically found in Zr alloys^7,28,30^. Figure 3 also shows that while the Zr-based materials have the lowest magnetic susceptibilities, the Ti-based materials are the second lowest magnetic susceptibilities. Nonetheless, Ti-6Al-4V and cp Ti-Gr2 still exhibit values almost three or two times larger than the Zr-based materials, consistent with previous investigations^7,10,11,17,28,30^. Based on these promising results, it is usually claimed that the reduced magnetic susceptibilities of Zr-based materials could sharply reduce the occurrence of MRI artifacts. However, they have never been tested and compared with other metallic materials in real clinical MRI practice.

**Figure 4:**
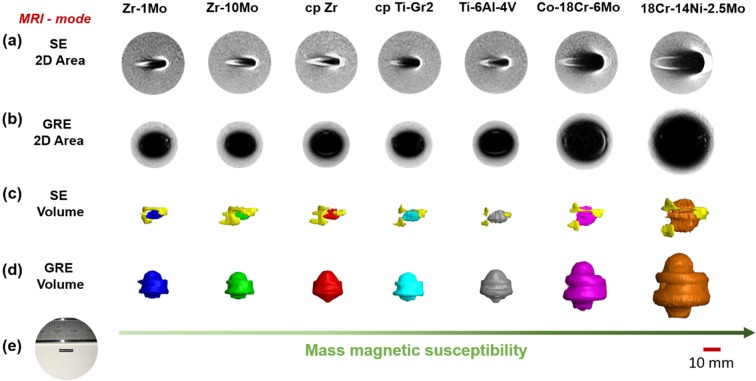
MRI of the Zr-based and other commercial metallic biomaterials: (**a**,**b**) 2D artifacts observed for SE and GRE pulse sequences, respectively; (**c**,**d**) 3D artifacts observed for SE and GRE pulse sequences, respectively; and (**e**) typical analysed sample. For interactive visualization click on the 3D PDF models in the Supplementary Material.

**Figure 5:**
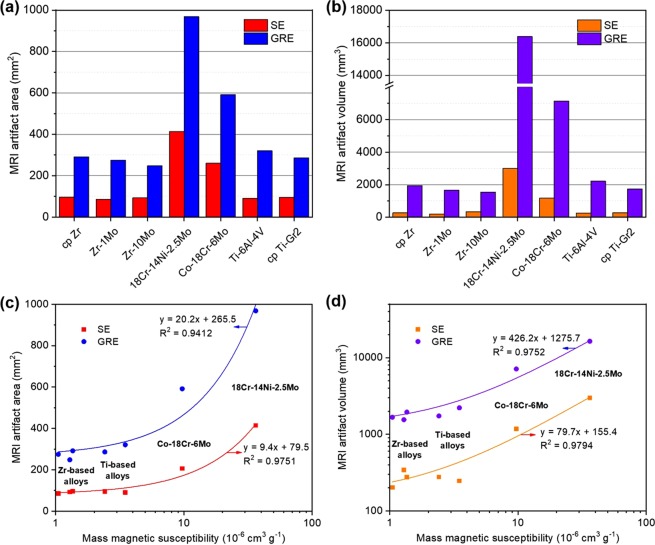
MRI of the Zr-based and other commercial metallic biomaterials: (**a**,**b**) measured artifact areas and volumes, respectively; (**c**,**d**) magnetic susceptibility versus artifact area and volume correlations, respectively.

Figure 4 shows the MRI performance of all the metals evaluated during this study. Surprisingly, we did not observe the expected magnetic susceptibility dependence when comparing artifacts of Zr- and Ti-based materials. For instance, Fig. 4a,b depict the 2D artifact appearance of the metallic samples for both spin echo (SE) and gradient echo (GRE) pulse sequences, respectively. It is noteworthy that excluding the 18Cr-14Ni-2.5Mo stainless steel and Co-18Cr-6Mo alloy, no clear difference can be observed. The same can be pointed out for the 3D artifacts depicted in Fig. 4c,d for the SE and GRE pulse sequences, respectively. Although some morphological differences can be verified among the artifacts of Zr- and Ti-based materials, their volumes seem to be very similar. A more detailed analysis is provided in Fig. 5a,b, which present the measured artifact areas and volumes, respectively. At first glance, the trend marked in these graphics resembles the previous trend for magnetic susceptibility (Fig. 3). However, a more careful look shows that no obvious trend exists between the results generated by the Zr- and Ti-based metals. For the SE sequence, the measured areas were 96.0, 85.3, 92.6, 414.3, 260.3, 90.4, and 95.3 mm^2^ for cp Zr, Zr-1Mo, Zr-10Mo, 18Cr-14Ni-2.5Mo stainless steel, Co-18Cr-6Mo, Ti-6Al-4V, and cp Ti-Gr2 materials, respectively. Consistent with results published in literature^21,25^, the areas observed for the GRE sequence were much larger: 291.3, 274.8, 248.1, 967.9, 591.1, 320.4, and 286.0 mm^2^ for Zr, Zr-1Mo, Zr-10Mo, 18Cr-14Ni-2.5Mo stainless steel, Co-18Cr-6Mo, Ti-6Al-4V, and cp Ti-Gr2, respectively. If these areas are plotted against the measured magnetic susceptibilities, an overall good linear relationship can be obtained (Fig. 5c), as reported by Imai *et al*.^25^. Nonetheless, there is a clear variability of the Zr and Ti points in relation to the fitted curves, showing that the studied Zr-based materials could not provide effectively lower artifact levels in comparison to the studied Ti-based materials despite their lower magnetic susceptibilities. For instance, if the lowest artifact areas for Zr- and Ti-based samples are compared, the reduction in the artifact area is only 5.6% for a reduction of  69.9% in the magnetic susceptibility using the SE sequence, and it is 13.3% for a reduction of 46.7% in the magnetic susceptibility using the GRE sequence. In the case of the 18Cr-14Ni-2.5Mo stainless steel and Co-18Cr-6Mo alloys, the artifact differences compared to Zr- and Ti-based metals are very prominent because of their much larger magnetic susceptibilities. As expected, the correlation between the calculated volumes and the measured magnetic susceptibilities (Fig. 5d) is consonant with the previous result (Fig. 5c), leading to a similar analysis. Based on these observations, it is possible to conclude that the magnetic susceptibility should vary significantly to provide relevant improvements in MRI. Therefore, it is believed that when the magnetic susceptibilities are relatively close, non-susceptibility-related artifacts, which are dependent, for example, on the size and orientation of the sample^31^, can significantly affect the overall MRI quality and cause deviations from the normally linear relationship between the magnetic susceptibility and the artifact area or volume. More studies are now required because this investigation was limited to samples with only one size and shape, as well as their orientation with respect to the main magnetic field was kept constant. Hence, it is important to verify whether similar conclusions would also be drawn by using different geometric features and under other experimental conditions.

In summary, this investigation showed that cp Zr, Zr-1Mo, and Zr-10Mo are favourable materials for biomedical and dentistry applications. Based on their mechanical properties, they can compete with typical metals in current usage; the Zr-10Mo alloy is particularly promising because of its relatively low Young’s modulus (~75 GPa). These alloys also presented low magnetic susceptibilities, which is an important property for MRI. In comparison with the cp Ti-Gr2, the Zr-based materials exhibited approximately half of its measured magnetic susceptibility. However, this did not translate effectively into smaller artifacts during MRI. Both Zr- and Ti-based metals produced similar artifacts without any obvious correlation between the measured artifact area or volume and the magnetic susceptibility. It was concluded that the magnetic susceptibility must vary significantly to obtain a noticeable enhancement, since non-susceptibility-related artifacts can significantly affect the overall MRI quality when comparing materials with relatively close magnetic susceptibilities.

## Methods

Mo was chosen as a viable alloying element because it is a strong β-stabilizer, has low cytotoxicity^32^, and low magnetic susceptibility^11,25^. The Zr and its alloys were arc-melted in an inert atmosphere using nuclear grade Zr (>99%, residual levels of Hf <5000 ppm and Fe <1000 ppm) and pure Mo (99.95%). The as-cast ingots were homogenized at 1000 °C for 12 h and then hot-rolled at 800 °C until a height reduction of approximately 60% was achieved. Thereafter, a solution heat treatment was applied at 1000 °C for 1 h, followed by furnace cooling. This processing route enabled favourable relationship between the mechanical properties (this will be discussed in a subsequent publication). The microstructures produced in the samples were characterized using visible-light microscopy and X-ray diffraction (XRD). Chemically polished samples (Kroll solution) were observed by a Leica DM IL LED microscope, while XRD was carried out via a PANalytical X’Pert PRO instrument equipped with CuKα radiation. The Young’s moduli were determined by ultrasonic measurements employing a Panametrics-NDT 5072PR pulser-receiver and piezoelectric transducers operating at 5 MHz^13,14^. For the following experiments, the Zr-based samples as well as commercial 18Cr-14Ni-2.5Mo stainless steel, Co-18Cr-6Mo, Ti-6Al-4V, and cp Ti-Gr2 samples were evaluated. Mechanical compression tests were performed at 2 mm min^−1^ at room temperature in an MTS 810 universal testing machine using three specimens of each alloy, 2 mm in diameter and 4 mm long. The magnetic susceptibility values were determined at room temperature using a Quantum Design PPMS system and applying a magnetic field of 0.35 T. Finally, MRI was performed at a static field strength of 1.5 T in a Toshiba Vantage machine using a standard head coil. Cylindrical specimens with a diameter of 2 mm and a length of 8 mm were embedded in commercial gelatine and thereafter positioned in the MRI scanner with their longitudinal axis aligned with the main magnetic field direction (z direction). Several slices were taken to cover the entire artifact extension using spin echo (SE) and gradient echo (GRE) pulse sequences. For the SE images, the parameters included a repetition time (TR) of 500 ms, echo time (TE) of 20 ms, and flip angle of 90°; for the GRE images, a TR of 500 ms, TE of 15 ms, and flip angle of 30° were employed. The slice with the largest artifact for each material and in each condition was selected and analysed with ImageJ software^33^ to determine the artifact area observed in the sagittal plane. The artifact area was estimated by pixels that deviated from the mean window level by more than ±30% of the free-artifact region of the image (background), similar to the procedure described in detail by Kawabata *et al*.^31^. Lastly, the artifact volume was estimated using a medical imaging program for 3D reconstruction and the SolidWorks engineering software. The RMI DICOM files were processed using the InVesalius^34^ software aimed to reconstruct and create STL files. All 3D surface files were created using the same threshold parameters (i.e., 0 = dark and 10000 = light, grey level). As SE images exhibit light and dark artifacts, two surface files were obtained (thresholds between 0–3000 for dark and 8000–10000 for light artifacts). GRE 3D surfaces were obtained using thresholds between 0–3000 for dark grey levels. Afterwards, SolidWorks was used to assembly the STL files, to calculate the total volume for both SE and GRE files, and to generate 3D PDF interactive files.

## Supplementary information

Supplementary information
