# Supplementary material for: Appraising the potential of Zr-based biomedical alloys to reduce magnetic resonance imaging artifacts
Source: Sci Rep. 2020 Feb 14;10:2621. doi: 10.1038/s41598-020-59247-1 (PMC7021671; doi:10.1038/s41598-020-59247-1)
Supplement: Supplementary file 1 — Supplementary information [file 41598_2020_59247_MOESM1_ESM.pdf]

Appraising the potential of Zr-based biomedical alloys to reduce magnetic resonance imaging artifacts

Anderson Kiyoshi Suzuki[a], Kaio Niitsu Campo[a], Eduardo Bertoni Fonseca[a], Luana Caldeira Araújo[a], Flávio César Guimarães Gandra[b], Éder Sócrates Najar Lopes[a*]

[a]School of Mechanical Engineering, University of Campinas – UNICAMP

13083-860, Campinas, SP, Brazil

[b]Institute of Physics “Gleb Wataghin”, University of Campinas – UNICAMP

13083-970, Campinas, SP, Brazil

* Corresponding author:
Phone: +55-19-35210017
Rua Mendeleyev, 200
Campinas, SP, 13083-860 Brazil
Email: ederlopes@fem.unicamp.br

S.1. Interactive visualization of Zr-1Mo alloy in RMI spin echo (SE) pulse sequence (3D PDF model).

S.2. Interactive visualization of Zr-1Mo alloy in RMI gradient echo (GRE) pulse sequence (3D PDF model).

S.3. Interactive visualization of Zr-10Mo alloy in RMI spin echo (SE) pulse sequence (3D PDF model).

S.4. Interactive visualization of Zr-10Mo alloy in RMI gradient echo (GRE) pulse sequence (3D PDF model).

S.5. Interactive visualization of cp Zr alloy in RMI spin echo (SE) pulse sequence (3D PDF model).

S.6. Interactive visualization of cp Zr alloy in RMI gradient echo (GRE) pulse sequence (3D PDF model).

S.7. Interactive visualization of cp Ti-Gr2 alloy in RMI spin echo (SE) pulse sequence (3D PDF model).

S.8. Interactive visualization of cp Ti-Gr2 alloy in RMI gradient echo (GRE) pulse sequence (3D PDF model).

S.9. Interactive visualization of Ti-6Al-4V alloy in RMI spin echo (SE) pulse sequence (3D PDF model).

S.10. Interactive visualization of Ti-6Al-4V alloy in RMI gradient echo (GRE) pulse sequence (3D PDF model).

S.11. Interactive visualization of Co-18Cr-6Mo alloy in RMI spin echo (SE) pulse sequence (3D PDF model).

S.12. Interactive visualization of Co-18Cr-6Mo alloy in RMI gradient echo (GRE) pulse sequence (3D PDF model).

S.13. Interactive visualization of 18Co-4Ni-2.5Mo alloy in RMI spin echo (SE) pulse sequence (3D PDF model).

S.14. Interactive visualization of 18Co-4Ni-2.5Mo alloy in RMI gradient echo (GRE) pulse sequence (3D PDF model).
